# Is it safe and effective to perform stapes surgery in Bellucci type 1 chronic otitis media?

**DOI:** 10.1007/s00405-025-09317-9

**Published:** 2025-03-28

**Authors:** Zafer Ciftci, Ibrahim Erdim, Hilmi Cem Kaya, Ismail Hologlu

**Affiliations:** 1 Hamidiye Faculty of Medicine, Department of Otorhinolaryngology, University of Health Sciences, Istanbul, Turkey; 2 Department of Otorhinolaryngology, Medipol University, Istanbul, Turkey

**Keywords:** Bellucci, Chronic otitis media, Otosclerosis, Stapedotomy

## Abstract

**Purpose:**

The study’s objective was to evaluate the safety and outcomes of stapes surgery in patients with Bellucci type 1 chronic otitis media.

**Methods:**

The study group was composed of Bellucci type 1 chronic otitis media patients who were found to have stapedial footplate fixation during surgery for chronic otitis media. Stapedotomy and prosthesis insertion were performed during the same operation. The control group consisted of patients with an intact tympanic membrane who underwent stapedotomy for primary otosclerosis. The study and control groups comprised 11 and 16 patients, respectively. No preoperative or intraoperative perforation was present in the control group. We recorded the pre-and post-operative air conduction thresholds, the presence and degree of any air-bone gap (ABG) and documented any postoperative complications. A comparison was then made between the two groups.

**Results:**

The mean preoperative air conduction threshold for patients in the study group was 51.73 dBHL, which improved to 33.27 dBHL after surgery. In the control group, the preoperative mean air conduction threshold was 50.13 dB HL, which also demonstrated significant improvement postoperatively, with a threshold of 29.5 dB HL. Preoperative ABG was 31.36 dBHL and 29.88 dBHL, respectively, in the study and control group. Postoperatively, ABG was reduced to 14.82 dBHL and 10.06dBHL in the study and control groups, respectively (*p* < 0.001).

**Conclusion:**

The preliminary findings of the present study indicated that concurrent tympanoplasty and stapedotomy in Bellucci type 1 chronic otitis media patients could yield safe and effective outcomes. Further investigations should be conducted to set forth the benefits of a single-stage surgery over a series of staged surgeries.

## Introductıon

Fixation of the stapedial footplate is a common finding in patients with chronic otitis media. This condition can be attributed to a tympano-sclerotic process affecting the ossicles, concurrent otosclerosis, or post-inflammatory osteogenic fixation of the stapes [1]. The traditional treatment for the disorder involves a series of staged surgeries. The surgical treatment for chronic otitis media, involving tympanoplasty, is performed in the first stage. In the second stage, the stapes footplate is fenestrated in a dry middle ear with an intact tympanic membrane. This staging approach aims to prevent post-operative infections and preserve sensorineural hearing [2, 3]. A similar approach is used when treating otosclerosis in patients who do not have chronic otitis media. If an unintended perforation of the tympanic membrane occurs during surgery, the stapes operation is postponed until the membrane has healed. To sum up, a perforation of the tympanic membrane - whether it is pre-existing or occurs during the surgery - is considered a contraindication for stapes surgery [4].

Despite traditional views, a recent paradigm shift in stapes surgery suggested it was likely safe to proceed with the procedure and perform concurrent tympanoplasty, even if an unintentional intraoperative perforation of the tympanic membrane occurs [5]. A recent animal study further proposed that treated pseudomonal otitis media did not increase the risk of sensorineural hearing loss in guinea pigs undergoing stapedectomy in an experimental setting [6]. To the best of our knowledge, the safety and outcomes of stapes surgery performed in dry human ears with chronic otitis media have not been previously evaluated. This study aims to examine the results of stapes surgery in patients with chronic otitis media who are classified under Group 1 of the Bellucci Dual Classification system [7]. We will also discuss the implications of our findings in the context of the existing literature.

## Materials and methods

The study was conducted in the otorhinolaryngology department of a tertiary care university hospital between April 2021 and September 2024. During this period, a total of 216 patients with Bellucci type 1 chronic otitis media underwent tympanoplasty and ossicular chain reconstruction. The study group was composed of Bellucci type 1 chronic otitis media patients who were found to have stapedial footplate fixation during surgery for chronic otitis media. Stapedotomy and prosthesis insertion were performed during the same operation. The control group consisted of patients with an intact tympanic membrane who underwent stapedotomy for primary otosclerosis. No preoperative or intraoperative perforation was present in the control group. The study and control groups comprised 11 and 16 patients, respectively.

All surgical operations were performed by the same surgeon. The mean hearing thresholds of the subjects were assessed at frequencies of 500, 1000, 2000, and 3000 Hz, as recommended by the American Academy of Otolaryngology– Head and Neck Surgery (AAO-HNS) [8]. Pre- and postoperative air conduction thresholds were recorded, along with the presence and degree of any air-bone gap (ABG). Postoperative hearing tests were conducted one year after the surgery. Additionally, postoperative complications were noted, and the two groups were compared.

### Statistical methods

Descriptive analyses were conducted to provide insights into the general characteristics of the study population. Shapiro-Wilk’s test assessed the normality of the variable distributions, and the results indicated that all variables exhibited a normal distribution. Consequently, a two-independent-sample t-test was utilized to compare the air conduction threshold and ABG between the study and control groups. To measure effect size, Cohen’s d coefficients were calculated. Continuous variables were presented as mean ± standard deviation, while categorical variables were compared using the Chi-Square test and reported as counts and percentages. A p-value of < 0.05 was considered statistically significant. All analyses were performed using commercial software (IBM SPSS Statistics, Version 23.0, Armonk, NY: IBM Corp.).

## Results

A total of 27 patients were enrolled in the research, which included 11 patients from the study group who underwent stapedotomy due to Bellucci type 1 chronic otitis media, and 16 patients from the control group who underwent stapedotomy for primary otosclerosis. The average ages of the study and control groups were 39.27 years and 41.38 years, respectively. The study group consisted of 5 male and 6 female patients, while the control group comprised 6 male and 10 female patients. In the study group, 6 patients had involvement of the left ear and 5 had involvement of the right ear. In the control group, 7 patients had left ear involvement and 9 had right ear involvement (Table 1).

**Table 1:** General characteristics of the patients

		Groups		*p*-value
		Chronic Otitis Stapedotomy (*n* = 11)	Otosclerosis Stapedotomy (*n* = 16)	
**Age**		39.27 ± 5,18	41.38 ± 4,9	0.294
**Gender**	**Male**	5 (45.5)	6 (37.5)	0.710
	**Female**	6 (54.5)	10 (62.5)	
**Side**	**Right**	5 (45.5)	9 (56.3)	0.873
	**Left**	6 (54.5)	7 (43.8)	

Statistics were showns as n (%) and mean ± standard deviation

**Fig. 1:**
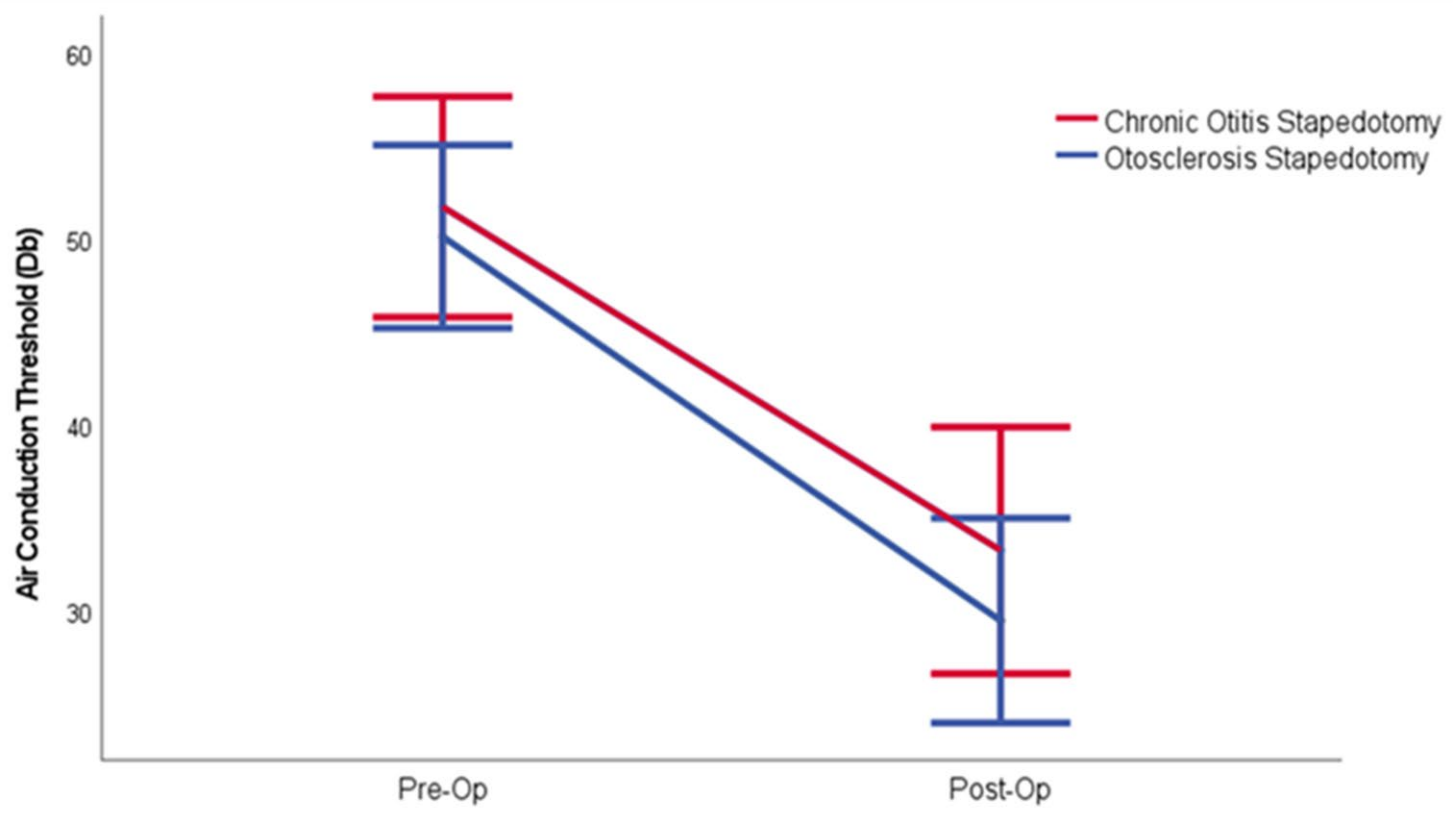
Comparison of the alteration between preoperative and postoperative air conduction thresholds in patients who underwent stapedotomy due to Bellucci Type1 Chronic otitis media and otosclerosis

The mean preoperative air conduction threshold for patients with chronic otitis media was 51.73 dB HL. In this group, the average air conduction threshold improved to 33.27 dB HL postoperatively, reflecting an improvement of 18.45 dB HL. In the second group, the preoperative mean air conduction threshold was 50.13 dB HL, which also showed significant improvement in the postoperative period, with a threshold of 29.5 dB HL. The differences between the pre- and postoperative air conduction thresholds for both groups were statistically significant (*p* < 0.001) (Table 2) (Fig. 1).

**Table 2:** Preoperative of postoperative air conduction thresholds in patients who underwent Stapedotomy due to Bellucci Type1 chronic otitis media and otosclerosis

		Groups		p-value	Effect Size (d)
		Chronic Otitis Stapedotomy (*n* = 11)	Otosclerosis Stapedotomy (*n* = 16)		
**Air Conduction Threshold (DbHL)**	**Preoperative**	51.73 ± 9.64	50.13 ± 9.46	0.672	0.168
	**Postoperative**	33.27 ± 9.78	29.5 ± 11.24	0.376	0.353
	**Differences**	18.45 ± 5.18	20.56 ± 4.68	0.281	-0.431
	**p-value**	**< 0.001**	**< 0.001**		

Statistics were shown as mean ± standard deviation

**Fig. 2:**
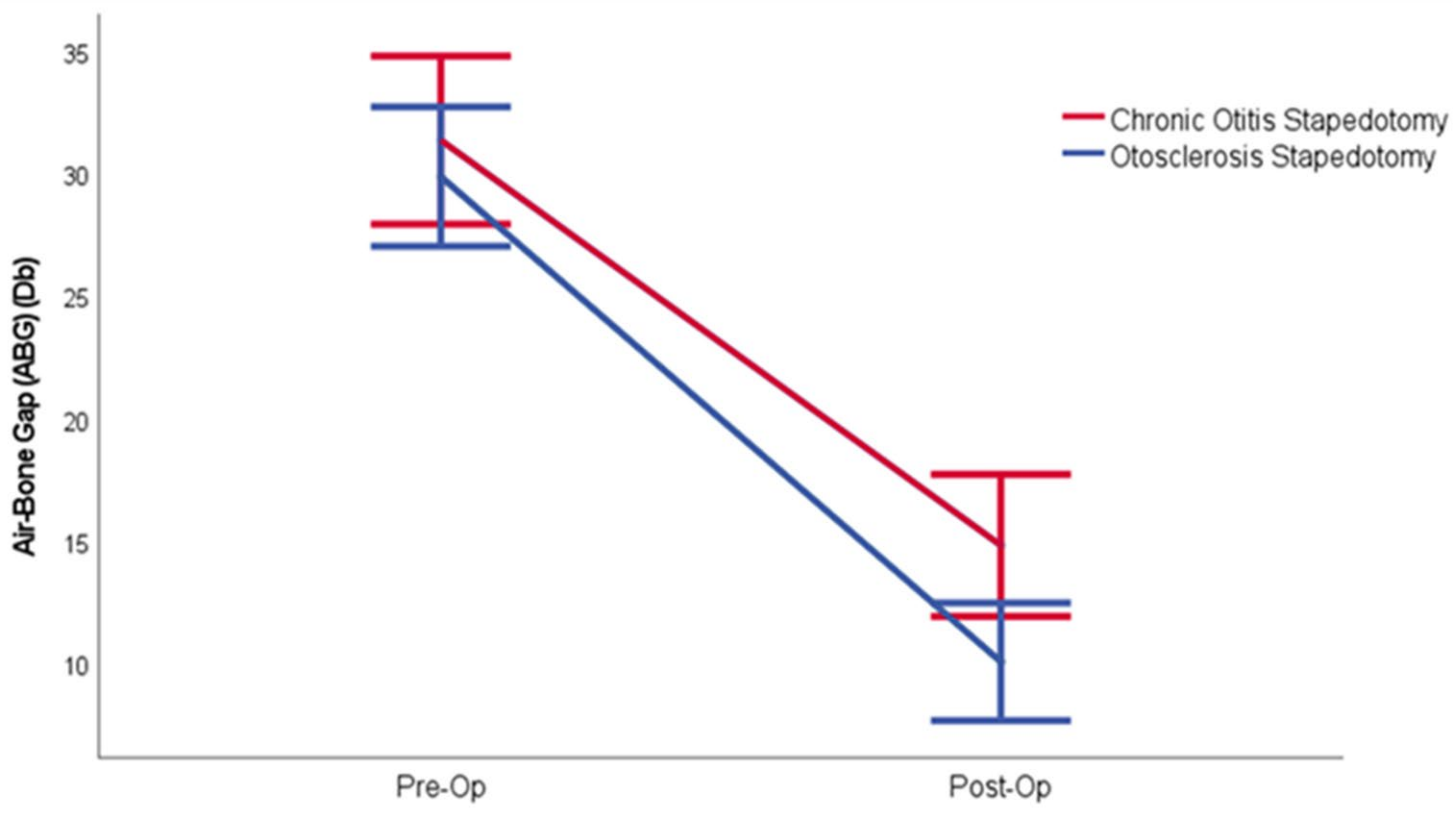
Comparison of the alteration between preoperative and postoperative air-bone gaps (ABG) in patients who underwent stapedotomy due to Bellucci Type 1 Chronic otitis media and otosclerosis

Preoperative ABG was 31.36 dBHL and 29.88 dBHL, respectively, in the study and control group. The ABG was reduced to 14.82 dBHL postoperatively in the study group. The postoperative ABG was found to be 10.06 dBHL in the control group. The difference in pre-and postoperative ABG values was statistically significant in both groups (*p* < 0.001) (Table 3) (Fig. 2).

**Table 3:** Preoperative of postoperative air-bone gaps (ABG) in patients who underwent Stapedotomy due to Bellucci Type1 chronic otitis and otosclerosis

		Groups		p-value	Effect Size (d)
		Chronic Otitis Stapedotomy (*n* = 11)	Otosclerosis Stapedotomy (*n* = 16)		
**Air-bone Gap (ABG)**	**Pre-Op**	31,36 ± 5,01	29,88 ± 5,84	0,498	0,270
	**Post-Op**	14,82 ± 3,16	10,06 ± 5,45	0,015	1,019
	**Differences**	16,55 ± 3,59	19,81 ± 4,42	0,053	-0,796
	**p-value**	**< 0.001**	**< 0.001**		

No sensorineural hearing loss was observed in both groups postoperatively. Similarly, none of the study and control group patients experienced vertigo symptoms or any signs of infection.

## Discussion

Staged surgeries have traditionally been the standard approach for treating stapedial footplate fixation associated with tympanic membrane perforations. In cases of chronic otitis media, the usual plan involves first performing a tympanoplasty to repair the eardrum and address any cholesteatoma or chronic ear issues. After a healing period of six to twelve months, a second-stage procedure, which involves stapedotomy and prosthesis insertion, is carried out once the ear is dry and properly aerated [9].

Intraoperative inadvertent perforations of the tympanic membrane that occur during surgery for primary otosclerosis are also considered a contraindication for continuing with stapes surgery. If such a perforation occurs, the surgeon is advised to terminate the stapes surgery and proceed with tympanoplasty in the same session. Likewise, a second-stage procedure should be planned once the tympanic membrane has completely healed [10]. Recent protocols recommend applying the principles mentioned above within the same context [11].

Despite the traditional approach that is widely accepted, we hypothesized that, based on the recent scientific publications and implicit suggestions of peer otologists, in selected cases of perforated dry ears with chronic otitis media and with strict adherence to appropriate antibiotic coverage, the presence of a tympanic membrane perforation, in a dry ear with chronic otitis media, may not necessarily warrant staging the surgery. Based on the findings of our preliminary study, we think that it may be a safe and effective option to perform a single-stage operation on this group of patients.

Recently, Luryi et al. published the results of their research, marking the first known review of inadvertent tympanic membrane perforation and hearing outcomes following stapes surgery. They suggested that it is “likely safe and appropriate to proceed with primary stapes surgery and concurrent tympanoplasty.” [5]. Our study also found that single-stage stapes surgery in patients with chronic otitis media did not significantly impact their hearing outcomes. Furthermore, in our research, the presence of tympanic membrane perforation was not found to be associated with an increased risk of postoperative infection and vertigo.

In a study conducted by Lo et al., the authors hypothesized that performing stapedotomy after treating otitis media would not increase the risk of sensorineural hearing loss [6]. In the experimental study, the middle ear cavities of guinea pigs were inoculated with Pseudomonas aeruginosa, and stapedotomies were performed after treating the middle ear infection. The researchers concluded that stapedotomy can be safely conducted in patients with treated otitis media. However, the researchers acknowledged that their experimental in vivo setting had limitations in accurately replicating the middle and inner ear conditions found in chronic otitis media. They also pointed out that the animal model used in their study did not adequately represent the full spectrum of the disease as it affects humans with chronic otitis media. The need to assess how human variables affect stapedotomy outcomes in chronic otitis media treatment was highlighted for further clinical studies. In this context, to our knowledge, the present study is the first research in the literature to investigate the outcomes of single-stage tympanoplasty and stapes surgery in patients with chronic otitis media who have a tympanic membrane perforation and dry middle ear mucosa.

Furthermore, there has long been a debate regarding the timing of tympanoplasty for repairing tympanic membrane perforations in chronic otitis media as a standalone issue. Despite differing opinions, Sheehy et al. proposed that the most significant factor in deciding whether to stage the tympanoplasty operation depends on the extent of mucous membrane disease [12]. We also believe that limiting the extent of mucosal involvement preoperatively through appropriate medical treatment and selecting ears with dry mucosa may lead to improved postoperative efficiency and safety regarding hearing thresholds and the risk of complications.

## Conclusion

The preliminary findings of the present study indicated that concurrent tympanoplasty and stapedotomy in Bellucci type 1 chronic otitis media patients could yield safe and effective outcomes. Further investigations should be conducted to set forth the benefits of a single-stage surgery over a series of staged surgeries for chronic otitis media patients who also have stapedial footplate fixation.
